# Observations on the Inoculation of Blennorhœa, (or Gonorrhœa) in Cases Where This Running Is Suddenly Suppressed, and the Suppression Attended with Dangerous Symptoms

**Published:** 1804-12-01

**Authors:** 


### Observations on the Inoculation of Blennorhœa, (or Gonorrhœa) *in Cases where this Running is suddenly suppressed, and the Suppression attended with dangerous Symptoms; by* Mr. Larrey.

THE author of this memoir relates several interesting cases, in which he effected a cure of serious maladies by the inoculation of the virus go orrhoicum, or a weak solution of ammonia in water.

Obs. I.* Many of the soldiers, while in Eygpt, were affected with very obstinate ophthalmies, attended with ulcerations of the eye-lids, which had a cancerous appearance. A fetid purulent humour run down, which excoriated that part of the cheek on which it rested, although but for a short time. The cornea was either perforated, or a staphylome appeared, and sometimes the membranes of the eyes received a carcinomatous character. These accidents never manifested themselves but in individuals who had been previously affected with gonnorrhœas. Mr. Larrey employed some general remedies, but chiefly the artificial or natural inoculation of gonorrhœa; the first of which was effected by injecting into the urethra a sufficiently strong alkaline solution, in order to produce a slight inflammation; in consequence of which, a new running ensued, and these gonnorrhœas have constantly removed ophthalmies of that nature.

Obs. II. In other cases, the suppression of these venereal runnings was followed by a copious secretion of the nasal mucus, which, from being in its natural state, without smell, whitish, and slightly saline, became greenish, liquid, and the smell of a gonorrhoic running. The membrana pituitaria excoriated; and when the disease was neglected, the ulceration became chancreous, the membrane was corroded, and the bones were affected The remedies employed in these affections were nearly th. same as those directed for gonorrhœas; but experience seems to prove, that the patient must be also treated with the internal use of mercurial preparations.

Obs. III. Some soldiers were affected with almost complete deafness, attended with vertigo, and very troublesome tinklings of the ear, in consequence of the suppression of gonnorhœa. Injections of different liquors into the meatus auditorius had been tried, and blisters applied in the vicinity, without any effect; on the contrary, the deafness seemed to increase. Mr. Larrey ordered injections of ammonia to be made into the urethra, which produced an irritation sufficient to re-establish the running; and from the first day of this running, the tinkling of the ear disappeared, the patients could hear much better, and distinguish different sounds. The cure was finished by the use of mercurial frictions, and some grains of muriat of mercury united with opium, and taken internally in a proper mixture. Another soldier was inoculated with the virus

* What we term *cases* the French surgeons call *observations*. Ed.

virus of a natural and fresh gonorrhœa, and when the running took place, the tinkling of the ear ceased; a few days after the patient could hear with the left ear, and by degrees was perfectly cured.

Obs. IV. A young lady had all the symptoms of a pulmonary consumption in the third stage; the expectoration was purulent, fetid, and greenish; the difficulty of breath and oppression very considerable. The odour and particular nature of the discharge brought up by coughing, caused the author to suspect the suppression of a leucorrhoic running. Upon inquiry, he found, that at the period when the disease had commenced, this lady had had a running of the vagina, which had been cured by injections of acetated lead and the use of some liquors; and that during the last four years, the breast had never ceased to be affected. Mr. Larrey no longer doubting the cause of this disease, injected a weak solution of volatile alkali at the entrance of the vagina, which immediately produced a considerable phlogosis, attended with a purulent running, which became, in the course of a few days, very copious. After this had continued twenty-four hours, the patient fell into a quiet sleep, without being troubled with cough and expectoration. The pains of the breast gradually abated, and a few days after she had scarcely any fever in the evening. While the running increased, the affection of the breast disappeared, and after a proper treatment, the appetite, force, and vigour of body returned.

Obs. V. A soldier was attacked with a purulent dysenteric flux, which he had suffered several years, and for which a great number of remedies had been unsuccessfully employed. The alvine excretions were frequent, often attended with tenesmus and violent colics, particularly during the night time. The patient was in a state of marasmus, when Mr. Larrey examined him; and learnt, that at the period when the dysenteric flux had commenced the patient had been affected with a gonorrhœa, which he had removed by astringent injections. The antisyphilitic treatment was then employed, and a few days were sufficient to produce a favourable alteration in the tsate of the disease; mercurial frictions on the belly seemed to be particularly serviceable, and he took internally corrosive sublimate. The vigour of his body began to return, and two months after the consultation, the patient attended to his usual business.

Obs. VI. Another soldier, aged 26, of a feeble constitution, was received into the hospital on account of a violent gonorrhœa By the use of sopient antispasmodic remedies medies, and the application of leeches, the most pressing symptoms were removed, and repose procured to the patient. The running increased, but it was fetid and greenish. Baths were continued, and also the use of a sopient emulsion; and he was directed to take an antisyphilitic potion in the morning. The running, however, remained copious, and continued so a month after his first entrance into the hospital. But about this period the patient imprudently used the cold bath; in consequence of which the running was suppressed, he became feverish, complained of pains in the sides, was obstinately constipated, had a burning sensation in the belly, ardor urinæ, and a great dryness of the skin. The following morning the whole surface of the body was affected with an erysipelatous inflammation, which, running through its stages, terminated on the 9th day in suppuration. This first began on the skin of the hands and feet; the epidermis was detached, and the suppuration so copious, that it required to be dressed four times a day with lint and cerate. The feverish symptoms disappeared, and this disease became rather idiopathic. The matter of the suppuration proved somewhat analogous to that of virulent gonorrhœas; it was thick, viscous, of a greenish colour, and emitted a fetid odour. It issued not only from all points of the skin, but also from the nostrils and cavity of the mouth. This state of general ulceration occasioned the greatest pains to the unfortunate patient, and he could not repose himself in any kind of posture. For more than a month after using the cold bath, ulceration had been as copious as general; during which period, exsiccatives had been applied, but the crust thus formed contained under it a greenish matter; the hair fell off, the nails were disorganised, they became thick, scaly, rough, and dark yellow. Mr. Larrey determined to put an end to this miserable state, by making injections into the urethra with the pus taken from the hands and feet; and the gonnorhœa soon appearing, the general suppuration diminished. The dressings with ceratum Saturni, and wine mixed with honey, were continued, and the patient took internally a sudorific rob with a little muriat of mercury, of ammonia, opium, and ether. The suppuration continued longer on the hands and feet than on the rest of the body, but at length the whole skin healed; and when no ulcerations were any longer perceived, Mr. Larrey ordered mercurial frictions to be applied every three or four days, and the use of baths. Towards the end of the treatment a bubo appeared, which opened of itself; the nails were reproduced; and the the patient left the hospital perfectly cured, five months after he had entered it.

Obs. VII. A soldier was sent to the hospital on account of a virulent gonnorrhœa, which he had had a few days. The matter that issued was greenish and fetid; he had violent pains the whole length of the urinary canal; the urine passed with difficulty, and with an insupportable burning sensation: the erections were frequent, and he was feverish and restless. By the use of sopient mucilaginous potions, baths, and very small dose of corrosive sublimate taken with milk, the symptoms greatly diminished, and the patient, complaining of nothing but the running, desired to be discharged from the hospital.— Some time after, this soldier, desirous of freeing himself from the running, took the advice of a quack, used the cold bath, and introduced into the urethra bougies covered with mercurial ointment. The gonorrhœa immediately ceased; but soon after a violent pain supervened in the right thigh, which obliged him again to have recourse to the hospital. The pain extended itself to the extremities, and last to the articulations of all the members, which became stiff and completely motionless. A fever afterwards supervened, attended with symptoms of mania. Mr. Larrey endeavoured to remove the most pressing complaints by venesection at the vena jugularis, by antispasmodic potions, pediluvia, and sinapisms applied to the soles of the feet; and though he succeeded in appeasing the disorder, the general pains remained almost the same. At length an injection of gonnorhoic liquor into the urethra was tried, by which the running returned; and as this became more copious, the symptoms diminished in proportion; so that after a fortnight they entirely disappeared. This second gonorrhœa was treated with mercurial preparations, in combination with antispasmodics. All the symptoms were gradually removed, and he left the hospital perfectly cured.

